# Energetic Butterfly: Heat-Resistant Diaminodinitro *trans*-Bimane

**DOI:** 10.3390/molecules24234324

**Published:** 2019-11-26

**Authors:** Pengcheng Zhang, Dheeraj Kumar, Lei Zhang, Daniel Shem-Tov, Natan Petrutik, Ajay Kumar Chinnam, Chuang Yao, Siping Pang, Michael Gozin

**Affiliations:** 1 School of Materials Science and Engineering, Beijing Institute of Technology, Beijing 100081, China; zhangpengchengxyz@163.com; 2 Department of Chemistry, Indian Institute of Technology Roorkee, Roorkee, Uttarakhand 247667, India; 3 Software Center for High Performance Numerical Simulation, and Laboratory of Computational Physics, Institute of Applied Physics and Computational Mathematics, Beijing 100088, China; zhang_lei@iapcm.ac.cn; 4 School of Chemistry, Faculty of Exact Sciences, Tel Aviv University, Tel Aviv 69978, Israel; dstov101@gmail.com (D.S.-T.); petro.natan@gmail.com (N.P.); ajay0802@gmail.com (A.K.C.); 5 Key Laboratory of Extraordinary Bond Engineering and Advance Materials Technology (EBEAM) of Chongqing, Yangtze Normal University, Chongqing 408100, China; yaochuang@yznu.cn

**Keywords:** DFT calculations, energetic materials, thermostable explosives and explosophore

## Abstract

Due to a significant and prolific activity in the field of design and synthesis of new energetic molecules, it becomes increasingly difficult to introduce new explosophore structures with attractive properties. In this work, we synthesized a *trans*-bimane-based energetic material—3,7-diamino-2,6-dinitro-1*H*,5*H*-pyrazolo-[1,2-a]pyrazole-1,5-dione (**4**), the structure of which was comprehensively analyzed by a variety of advanced spectroscopic methods and by X-ray crystallo-graphy (with density of 1.845 g·cm^−3^ at 173 K). Although obtained crystals of **4** contained solvent molecules in their structure, state-of-the-art density functional theory (DFT) computational techniques allowed us to predict that solvent-free crystals of this explosive would preserve a similar tightly packed planar layered molecular arrangement, with the same number of molecules of **4** per unit cell, but with a smaller unit cell volume and therefore higher energy density. Explosive **4** was found to be heat resistant, with an onset decomposition temperature of 328.8 °C, and was calculated to exhibit velocity of detonation in a range of 6.88–7.14 km·s^−1^ and detonation pressure in the range of 19.14–22.04 GPa, using for comparison both HASEM and the EXPLO 5 software. Our results indicate that the *trans*-bimane explosophore could be a viable platform for the development of new thermostable energetic materials.

## 1. Introduction

**Figure 1:**
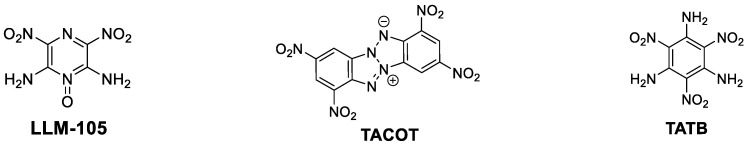
Structures of LLM-105 (2,6-diamino-3,5-dinitropyrazine-1-oxide), TACOT (1,3,7,9-tetranitro-benzo[*d*]-benzo [4,5][1,2,3]triazolo[2,1-*a*][1,2,3]triazol-11-ium-6-ide), and TATB (2,4,6-triamino-1,3,5-trinitrobenzene) thermostable explosives.

The development of new heat-resistant energetic materials (EMs) with improved thermostability is of great interest for a broad range of civil and defense applications. For example, in the aerospace industry, thermostable EMs are essential for providing unique solutions for stringent requirements for reliable and safe space-craft stage-separations and gear deployment [1,2,3]. In the case of deep-well oil and gas drilling, perforating guns, based on thermostable EMs, ensure efficient penetration and easier access to soil-embedded deposits at great depth, where temperatures may reach above 250 °C [4,5]. Designing EMs that would fit the criteria of thermostability (temperature of decomposition, T_d_ > 250 °C) imposes certain molecular and crystal architectures [6]. In recent years, several innovative molecular design strategies were introduced to obtain EMs with improved thermostability, which are based on advanced understanding of the relationships between molecular and crystal structures of an explosive and its properties [7]. There are few major design approaches that can lead to improved thermostability of EM: (i) vicinity of amino and nitro groups, positioned on an unsaturated bond in EM’s molecular structure, for example in 2,6-diamino-3,5-dinitropyrazine-1-oxide (LLM-105, T_onset_ = 298 °C, T_d_ = 341 °C) [8]; (ii) “heat-diffusing” molecular structures of EMs, based on incorporation of conjugated, aromatic moieties and fused-rings structures, as in 1,3,7,9-tetranitro-benzo[*d*]-benzo [4,5][1,2,3]triazolo[2,1-*a*][1,2,3]triazol-11-ium-6-ide (z-TACOT, T_d_ = 378 °C, y-TACOT, T_d_ = 400 °C) [9]; and (iii) molecular π–π stacking, hydrogen bonding and other intermolecular interactions in crystal structures of EMs, as in case of 2,4,6-triamino-1,3,5-trinitrobenzene (TATB, T_onset_ = 330 °C) (Figure 1) [10].

**Figure 2:**
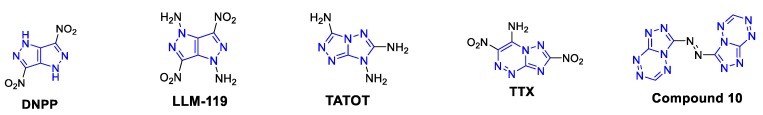
Structures and explosophore backbones of DNPP (1*H*,4*H*-3,6-dinitropyrazolo[4,3-c]pyrazole), LLM-119 (1,4-diamino-3,6-dinitropyrazolo[4,3-c]-pyrazole), TATOT (3,6,7-tri-amino-[1,2,4]triazolo[4,3-b][1,2,4]triazole), TTX (4-amino-3,7-dinitro-[1,2,4]triazolo[5,1-c] [1,2,4]triazine), and compound **10** thermostable explosives.

One of the promising molecular design strategies for the preparation of thermostable EMs is a fused-rings approach, which was utilized for the construction of a series of explosophores having a 1,4-dihydropyrazolo[4,3-c]-pyrazole backbone, as in 1*H*,4*H*-3,6-dinitropyrazolo[4,3-c]pyrazole (DNPP, T_d_ = 330 °C) [11] and 1,4-diamino-3,6-dinitropyrazolo[4,3-c]-pyrazole (LLM-119, T_d_ = 253 °C) [12]. It was also used in the design of 3,6,7-tri-amino-[1,2,4]triazolo[4,3-b][1,2,4]triazole (TATOT, T_d_ = 245 °C) [13] that has a [1,2,4]triazolo[4,3-b][1,2,4]-triazole backbone; in TACOT, with a 1*H*,5*H*-[1,2,3]triazolo-[2,1-a][1,2,3]triazole backbone; in 4-amino-3,7-dinitro-[1,2,4]triazolo[5,1-c] [1,2,4]triazine (TTX, T_d_ = 272 °C) [14], having a [1,2,4]triazolo[5,1-c][1,2,4]triazine backbone and in 1,2-bis([1,2,4]triazolo[4,3-b][1,2,4,5]tetrazine-3-yl) diazene (compound **10**, T_d_ = 305 °C) [15] explosive, having a [1,2,4]triazolo[4,3-b][1,2,4,5]tetrazine backbone (Figure 2).

**Figure 3:**
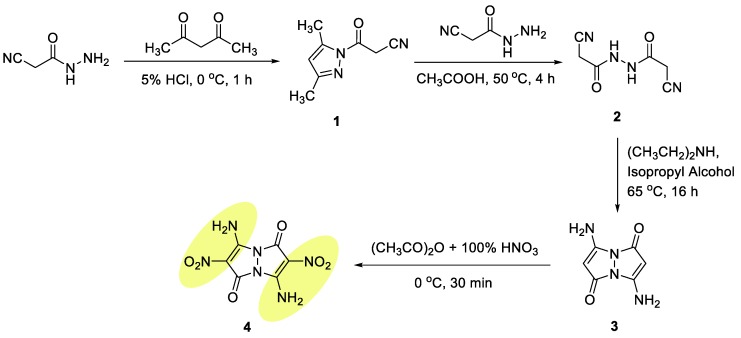
Synthesis of compound **4**.

Despite a great variety of explosophores based on five- and six-membered fused-heterocyclic rings, EMs containing two N–N-fused pyrazole rings in their backbone—1*H*,5*H*-pyrazolo[1,2-a]pyrazole-1,5-dione (*anti*-bimane) remained unexplored. Although low-yield synthesis of 3,7-diamino-2,6-dinitro-1*H*,5*H*-pyrazolo[1,2-a]-pyrazole-1,5-dione (**4**, Figure 3) was mentioned by Boyer and coworkers [16], no evaluation of this compound’s energetic properties was conducted. In this work, we report significantly improved synthetic protocols for the preparation of compound **4**, its comprehensive structural characterization, study of its thermal and detonation properties, as well as evaluation of its sensitivity to mechanical impact, friction, and electrostatic discharge. We also conducted density functional theory (DFT) calculations, to predict the molecular arrangement and structure of solvent-free crystals of **4**.

## 2. Results and Discussion

### 2.1. Synthesis

The target 3,7-diamino-2,6-dinitro-1*H*,5*H*-pyrazolo[1,2-*a*]pyrazole-1,5-dione **4** EM was prepared in four steps, starting with the synthesis of 3-(3,5-dimethyl-1*H*-pyrazol-1-yl)-3-oxopropanenitrile (**1**) with a 94% yield, via reaction of 2-cyanoacetohydrazide with acetylacetone under aqueous acidic conditions (Figure 3) [17]. A mono-cyclization reaction was followed by transformation of compound **1** into the corresponding 2-cyano-*N*′-(2-cyanoacetyl)-acetohydrazide intermediate **2** with a 92% yield, by heating of compound **1** with 2-cyanoaceto-hydrazide in glacial acetic acid. In a subsequent step, intermediate **2** was cyclized into 3,7-diamino-1*H*,5*H*-pyrazolo[1,2-*a*]-pyrazole-1,5-dione bimane **3** with a 78% yield. Although the latter cyclization reaction was vaguely reported by Schmidt and Schoenafinger [18], it indicated a maximum reaction yield of only 35%. The desire for a much higher yield led us to extensively study and develop optimized reaction conditions, which included the use of diethylamine, as a base; isopropanol, as solvent; and setting the reaction temperature at 65 °C. The synthesis of the target EM **4** was completed by nitration of 3,7-diamino bimane **3** with a 91% yield, utilizing a mixture of concentrated nitric acid and acetic anhydride at 0 °C.

We found that compound **4** has very poor solubility in water and most organic solvents, except DMF and DMSO, which may result in a good toxicological profile for this EM. All attempts to convert compound **4** into corresponding 2,3,6,7-tetranitro-1*H*,5*H*-pyrazolo[1,2-a]-pyrazole-1,5-dione via preparation of 2,6-dinitro-1,5-dioxo-1*H*,5*H*-pyrazolo[1,2-a]pyrazole-3,7-bis-(diazonium) salt or via oxidation of amine groups of **4**, were not successful under all explored reaction conditions. Further experiments to obtain *N*,*N*′-(2,6-dinitro-1,5-dioxo-1*H*,5*H*-pyrazolo[1,2-*a*]-pyrazole-3,7-diyl)-dinitramide derivative of **4** were also not successful, and under all examined conditions only starting 3,7-diamino-2,6-dinitro bimane **4** was recovered, or under more drastic temperature regimes, decomposition of the *trans*-bimane frame took place.

### 2.2. Spectral Studies

The targeted energetic 3,7-diamino-2,6-dinitro-1*H*,5*H*-pyrazolo[1,2-*a*]pyrazole-1,5-dione (**4**), as well as its intermediates were comprehensively characterized by ^1^H- and ^13^C-NMR, mass spectrometry, infrared spectroscopy, and elemental analysis (SI). ^1^H-NMR spectra of energetic compound **4** showed two sharp peak signals at 9.61 and 9.47 ppm corresponding to NH_2_ protons, which are downfield shifted, compared to NH_2_ protons in its precursor—3,7-diamino-1*H*,5*H*-pyrazolo[1,2-*a*]pyrazole-1,5-dione (**3**), which were observes with peak signals at 7.45 ppm. This can be attributed to the fact that in each of the NH_2_ groups in compound **4**, one of the protons is involved in the intra-molecular hydrogen bonding with the neighboring nitro groups, resulting in two different, but rather close proton peak signals. The resultant ^13^C-NMR spectra of compound **4** showed three signals at 153.5, 151.1, and 107.6 ppm. The signal corresponding to the carbonyl carbon in compound **4** at 153.5 ppm was found to be upfield shifted compared to the carbonyl carbon in compound **3** measured at 165.5 ppm. A similar observation was measured for the signals corresponding to the amino-substituted carbon in **3** (159.3 ppm) and **4** (151.1 ppm), while signal corresponding to the nitro-substituted carbon in the energetic compound **4** was found to be the most shielded, at 107.6 ppm.

### 2.3. X-ray Crystallography

**Figure 4:**
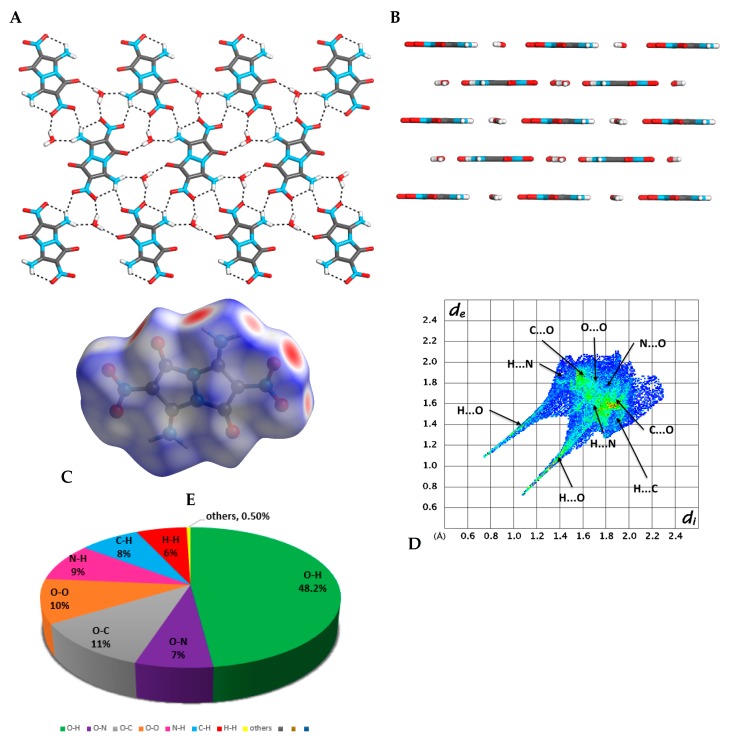
Analysis of crystal structure of compound **4**. (**A**) A view from above of a single-layer arrangement of molecules of **4**, and hydrogen bonding within the plane; (**B**) a layered arrangement of molecules of compound **4** in its crystal; (**C**) a Hirschfeld surface analysis of compound **4** in its crystal structure; (**D**) a fingerprint plot of **4** presenting the contribution of all significant intermolecular interactions; (**E**) pie-type presentation of individual atomic contact percentage contribution to the Hirschfeld surface.

Crystals of compound **4** (CCDC 1955362), suitable for X-ray crystallography, were obtained from an aqueous DMSO or DMF solutions. Compound **4** was crystalized in the form of yellow plate crystals (with calculated density of 1.845 g·cm^−3^ at 173 K), in the monoclinic space group P21/c, containing five molecules of **4** and four molecules of water in a single unit cell, forming a plane-layered stacked structure (Figure 4A,B) (see Supplementary Materials Tables S1–S5). This crystal arrangement is held together by intermolecular interactions between molecules of **4** and molecules of water. Each molecule of water interacts with three adjacent molecules of **4** via in-plane intermolecular hydrogen interactions (Figure 4A), and with one molecule of **4** in a parallel plane via π-type interactions (Figure 4B). The intermolecular and intramolecular hydrogen bonding between the NO_2_ and the NH_2_ groups are responsible to the fixation of the NO_2_ groups in the plane of the molecule, which could be one of the main reasons for compound **4**’s relatively low friction sensitivity [19] (Figure 4A). Figure 4C shows the calculated Hirschfeld surface of molecule **4** in its crystal structure (Figure 4C), presenting the contribution of all intermolecular interactions and atom-specific non-covalent intermolecular interactions (cohesive free energy) in this molecule (Figure 4D). The appearance of the red spots on the Hirschfeld surface and thick spikes in the corresponding fingerprint plot (Figure 4D) indicates a high influence of the in-plane intermolecular hydrogen bonds on the crystal packing of compound **4**, where the short *d_i_* + *d_e_* values clearly indicate that these hydrogen bonds are very strong. The Hirschfeld surface shows that O···H interactions have the highest influence (48.2%) on the arrangement of molecule **4** in its crystal (Figure 4E). As could also be seen from the Hirschfeld surface, the hydrogen bond interactions between water molecules and the diamino-dinitro-*trans*-bimane have a significant influence (16.7%) on the 3D arrangement of molecules of **4** in its crystal, connecting the parallel sheet-type layers. The extensive hydrogen bonding network between the molecules of **4** in the crystal leads to their tight association, with a binding energy (BE) of 72.99 kcal·mol^−1^ and a packing coefficient (PC) of 80.02%.

### 2.4. DFT Structure Calculations

Taking the lattice parameters and atomic coordinates from single-crystal X-ray diffraction analysis as input, we optimized the crystal geometry on the basis of the conjugate gradient (CG) method [20]. The calculated structures were considered as finally optimized, when the residual forces were less than 0.03 eV/Å and the stress components were less than 0.01 GPa. The simulated structure showed satisfactory agreement with the characterized structure by X-ray diffraction, with the discrepancy of the volume at 0.8% (Table 1). Very small discrepancies between the calculated and the experimental values showed the reliability of the calculation method that was used in this work. 

**Figure 5:**
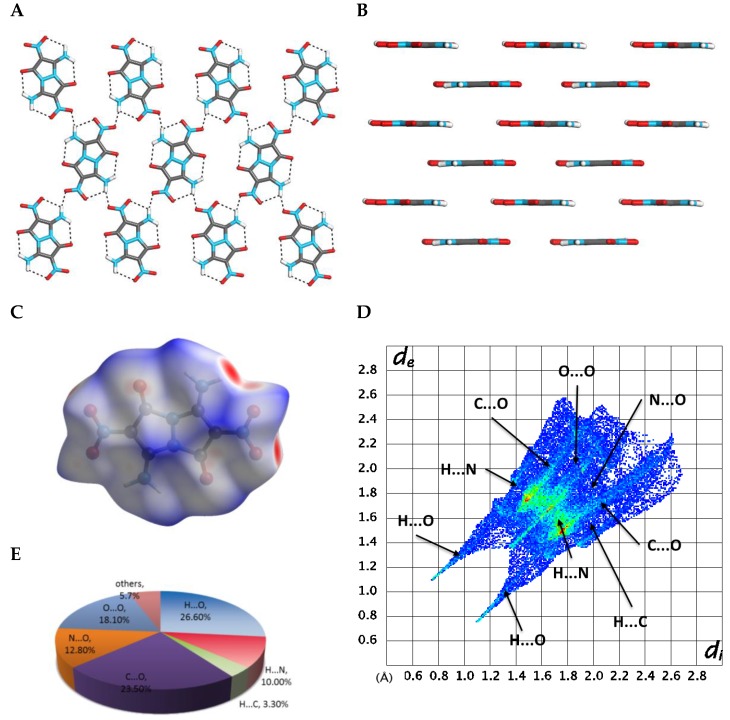
Analysis of calculated crystal structure of compound **4** from which water molecules were removed. (**A**) A view from above of a single-layer arrangement of molecules of **4**, and hydrogen bonding within the plane; (**B**) a layered arrangement of molecules of compound **4** in its crystal; (**C**) a Hirschfeld surface analysis of compound **4** in its crystal structure; (**D**) a fingerprint plot of **4** presenting the contribution of all significant intermolecular interactions; (**E**) pie-type presentation of individual atomic contact percentage contribution to the Hirschfeld surface.

In order to study the solvent-free crystal, we removed the water molecules and re-optimized the crystal structure by using CG method (Figure 5) [20]. The optimized calculated structures were then used for energetics analysis and physicochemical properties calculations. After removing the water molecules and re-optimizing the crystal structure of compound **4**, the symmetry of the crystal remained P21/c. However, all three lattice lengths shrunk, and the volume of the unit cell was reduced by 10%. Although the layer-by-layer stacking arrangement remained (Figure 5B), the in-layer hydrogen bond network was now constituted only from N–H···O bonds, formed between the molecules of **4** (Figure 5A). Comparing the water-free to the water-including structures of **4**, we found that the area of the red spots on the Hirschfeld surface was reduced, the spikes in the 2D fingerprint become more thinly distributed, while the *d_i_* + *d_e_* values became larger (Figure 5C,D). These changes suggest that presence of the water solvent in the crystal structure of **4**, makes this structure stronger and, thus, more thermodynamically stable. The hydrogen bonding attraction in the water-free structure possesses 39.9% of the total weak interactions (Figure 5E), which is 25% less in comparison to the water-including structure of **4**. In contrast, removal of water resulted in the significant increase in N···O and O···O intermolecular repulsive interactions (which are 30.9% of the amount of the total interactions), in comparison to the original water-including crystal structure of **4** (which has 17.3% of the N···O and O···O intermolecular repulsive interactions). All these changes in the water-free structure of **4** resulted in a smaller crystal packing force, which was reflected by a sharply reduced BE of 51.10 kcal·mol^−1^ and a smaller PC of 77.43%, versus BE of 72.99 kcal·mol^−1^ and PC of 80.02% of water-containing structure.

**Figure 6:**
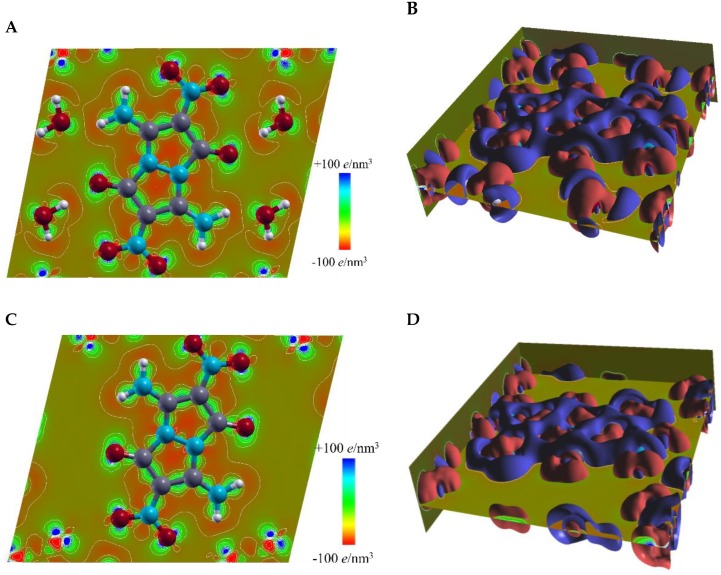
Calculated valence charge distribution in the plane of molecule **4**. (**A**) A top view of charge distribution in water-containing crystal structure of **4**; (**B**) calculated isosurface of the valence charge density at +8 *e*/nm^3^ (red surfaces) and −8 *e*/nm^3^ (blue surfaces) in water-containing crystal structure of **4**; (**C**) top view of charge distribution in water-removed crystal structure of **4**; (**D**) calculated isosurface of the valence charge density at +8 *e*/nm^3^ (red surfaces) and −8 *e*/nm^3^ (blue surfaces) in water-removed crystal structure of **4.**

To study water-containing and water-removed crystal structures of compound **4** from the electronic scale perspective, we plotted in real space the valence charge distribution in the plane of molecule **4**, with the neutral atomic charge densities set as the reference, as shown in the contour map of Figure 6A,C, and in the isosurface plot of Figure 6B,D. Referring to the system of separate atoms, electrons in the molecules in the crystal showed strong aggregation surrounding the chemical bonds, while exhibiting delocalization at the outer borders of the molecules. The deformation of the electronic structure of the molecules in the crystal was caused by crystal packing and intermolecular interactions. We calculated that in the water-removed structure, the electron aggregation around the chemical bonds became relatively weaker and the electron delocalization at the molecule borders occurred in a smaller region (Figure 6C,D), versus the water-containing structure of **4** (Figure 6A,B).

### 2.5. Physiochemical and Energetic Properties

**Figure 7:**
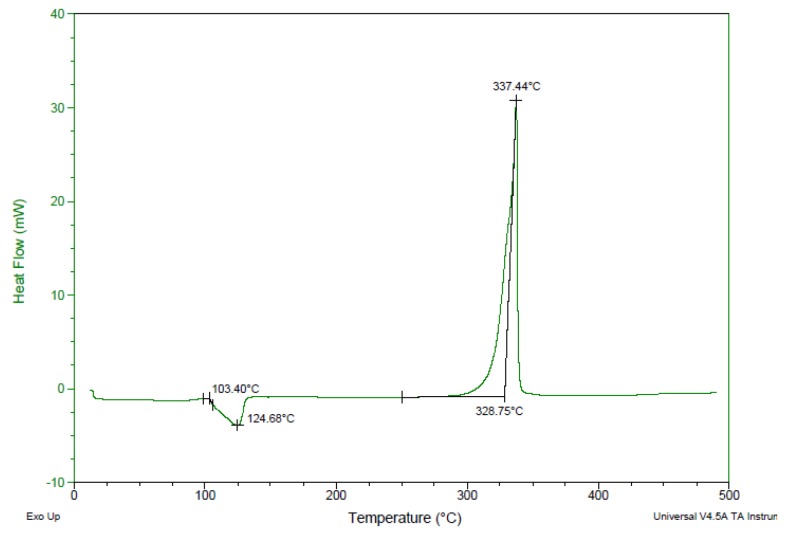
Differential scanning calorimetry (DSC) thermogram of compound **4** with an onset decomposition temperature of 328.8 °C.

The thermal behavior of compound **4** was studied by differential scanning calorimetry (DSC) at a heating rate of 5 °C·min^−1^ (Figure 7). Compound **4** exhibited an exothermic peak with onset decomposition temperature (T_d/onset_) of 328.8 °C. The thermal performance of compound **4** was found to be comparable to commonly used thermostable explosives, such as HNS (T_onset_ = 320 °C), TATB (T_onset_ = 330 °C), and LLM-105 (T_d/onset_ = 298 °C; peak decomposition temperature T_d/peak_ = 341 °C), suggesting the potential usability of compound **4** as a new highly thermostable EM.

The heat of formation of compound **4** was calculated by using isodesmic equations and the G4 software program and was found to be −181.131 kJ·mol^−1^ (Equation (1) and Table 2).

Δ_f_H°_gas_ (compound **4**) = 2 × Δ_f_H°_gas_ (CH_3_NO_2_) + 2 × Δ_f_H°_gas_ (CH_3_NH_2_) + 2 × Δ_f_H°_gas_ (CH_2_O) + Δ_f_H°_gas_ (C_6_H_8_N_2_) − 6 × Δ_f_H°_gas_ (CH_4_) − ∆E_0_ − ∆ZPE − ∆H_T_ = −181.13 kJ·mol^−1^ (1)

Applying the measured ambient temperature density and the calculated ΔH_**f**_ values into EXPLO 5 (v6.04) software, allowed us to predict the velocity of detonation (VOD) and the detonation pressure (P_*d*_) of compound **4** (Table 3). The VOD of **4** was calculated to be 7.72 km·s^−1^, while the P_*d*_ was calculated to be 24.33 GPa. These modest calculated detonation performance of compound **4** could be attributed to a very low negative ΔH_**f**_ of this compound (−707.18 kJ·kg^−1^), in comparison with the moderately low ΔH_**f**_ values reported for TATB (−596.55 kJ·kg^−1^), HNS (173.93 kJ·kg^−1^), and FOX7 (−905.42 kJ·kg^−1^).

**Figure 8:**
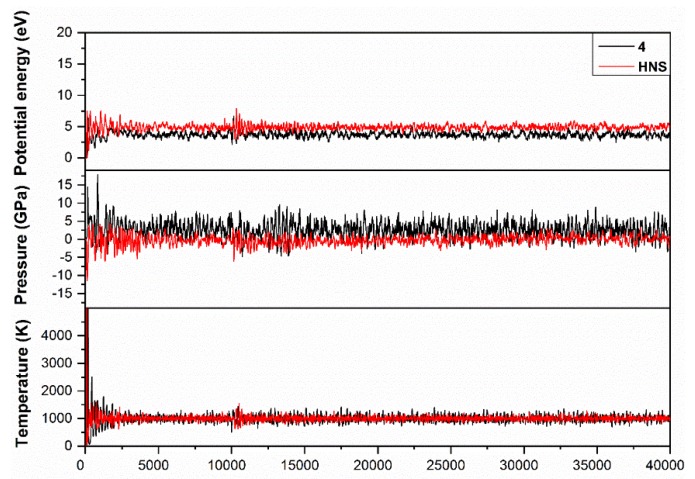
Potential energy, pressure, and temperature of compound **4** were calculated as a function of time during the ab initio molecular dynamics (MD) simulations, with HNS as reference compound.

In order to study the dynamics of the chemical bonds under heating stimulus, we performed ab initio molecular dynamics (MD) simulations with HASEM package, using the optimized structures of compound **4** and HNS as the reference explosive. A canonical (NVT) ensemble was employed to simulate the heating of the systems from 0 to 1000 K for 40,000 steps (time step set to be 0.2 fs). For each atomic iteration, the density matrix was considered as converged when smaller than 5.0 × 10^−6^ ē and the atomic force tolerance was set to be 0.04 eV/Å. As shown in Figure 8, the potential energy, pressure, and temperature of both systems converged from ~300 steps. From then on, the temperature of each system was constrained at around 1000 K, and the breaking and recombination of the weak chemical bonds occurred with the frequency dependent on their dissociation barrier. Within the simulation interval, each trigger C–NO_2_ bond in compound **4** broke and recombined 87.25 times on average, close to 80.46 times of the HNS reference. However, HNS could competitively initiate at an alternate location, i.e., C–C bridge bond, resulting in its slightly lower decomposition temperature than compound **4**, matching our experimental thermal analysis observations (Table 4).

The bulk-powder density of energetic compound **4** was measured by gas pycnometer at ambient temperature and found to be 1.88 g·cm^−3^, which is slightly lower than the densities of TATB, LLM-105, and higher than of HNS (Table 4). 

Evaluation of the sensitivity by standard BAM methods to impact was performed in accordance with NATO STANAG 4489; Explosives, Impact Sensitivity Tests. The sensitivity to friction was performed with accordance to NATO STANAG 4487; Explosives, Friction Sensitivity Tests. The sensitivity toward electrostatic discharge was performed with accordance to NATO STANAG 4490; Explosives, Electrostatic Discharge Sensitivity Tests.

## 3. Materials and Methods

### General Information

All chemical reagents and solvents used in this work were of analytical grade and were used as supplied, without additional purification. ^1^H and ^13^C-NMR spectra were recorded on a Bruker Avance III 400 MHz spectrometer at 25 °C (see Supplementary Materials Figures S1–S4, Figures S5–S8). Infrared spectra were measured on a Bruker Tensor 27 FTIR spectrometer equipped with a diamond ATR unit (see Supplementary Materials Figures S9–S12). Melting and decomposition temperatures were measured by differential scanning calorimetry at a scan rate of 5 °C·min^−1^. Densities were determined at room temperature on a Micromeritics AccuPyc 1330 gas pycnometer. The impact sensitivity of compounds was tested according to STANAG 4489 protocols, using a BAM drop-hammer. The friction sensitivity was tested according STANAG 4487 protocols, using a BAM friction tester.

Single-crystal X-ray diffraction measurements for compound **4** were performed on a Bruker Nonius Kappa Apex2 diffractometer, using graphite-monochromated MoKα radiation (λ = 175 mm^−1^). All diffractometer manipulations, including data collection, integration, scaling, and absorption corrections were carried out by using the Bruker Apex2 software. Data collection was carried out at 173 K, using a frame time of 20 s and a detector distance of 60 mm. The structure of compound **4** was solved by using SHELXS and refined by least-squares methods using SHELXL. The structures compound **4** were solved using SIR92 and subsequent electron-density difference syntheses and refined (full-matrix least-squares) using the Oxford University Crystals program. All non-hydrogen atoms were refined using anisotropic displacement parameters.

All the density functional theory (DFT) calculations were performed using the HASEM software [28]. The generalized gradient approximation method was used for the exchange-correlation functional in the Perdew–Burke–Ernzerhof form. Norm-conserving pseudo-potentials were used to replace the core electrons. The valence electrons are described by linear combinations of numerical pseudoatomic orbitals. The reliability of this method to predict the energies and structures of energetic molecular crystals has been demonstrated in previous work by comparison with CCSD(T) results and experimental measurements [29,30,31].

*3-(3,5-Dimethyl-1H-Pyrazol-1-yl)-3-Oxopropanenitrile* (**1**). To a solution of 2-cyanoacetohydrazide (10.0 g, 0.10 mol) in aqueous HCl (5% in H_2_O; 60 mL) neat acetylacetone (10.1 g, 0.10 mol) was added dropwise at 4 °C. At the end of the addition, the reaction mixture was stirred for an additional 1 h. The formed precipitate of crude product was filtered, washed with cold water (3 × 10 mL), and crystallized from CCl_4_ to afford pure 3-(3,5-dimethyl-1*H*-pyrazol-1-yl)-3-oxopropanenitrile (**1**) as a white solid (15.50 g, 94%). ^1^H-NMR (400 MHz, CDCl_3_): δ 6.01 (s, 1H, NH), 4.27 (s, 2H, CH_2_). 2.52 (s, 3H, CH_3_), 2.20 (s, 3H, CH_3_). ^13^C-NMR (100 MHz, CDCl_3_): δ 162.4, 153.8, 144.6, 113.4, 112.4, 26.8, 13.9, 13.7. IR (ATR, cm^−1^): 3406, 2974, 2940, 2899, 2264, 1731, 1585, 1479, 1387, 1365, 1310, 1246, 1139, 1012, 964, 926, 822, 750. Elemental analysis calc. (%) for C_8_H_9_N_3_O (163.18): C 58.88, H 5.56, N 25.75; found: C 58.75, H 5.65, N 25.51.

*2-Cyano-N′-(2-Cyanoacetyl)Acetohydrazide* (**2**). A mixture of 3-(3,5-dimethyl-1*H*-pyrazol-1-yl)-3-oxo-propanenitrile (**1**) (8.16 g, 0.05 mol) and 2-cyanoacetohydrazide (5.00 g, 0.05 mol) in glacial acetic acid (60 mL) was heated at 50 °C for 4 h. After that time, the reaction mixture was cooled down to room temperature, and the formed precipitate of a crude product was filtered, washed with acetic acid (3 × 10 mL), and vacuum dried to afford 2-cyano-*N*′-(2-cyanoacetyl)acetohydrazide (**2**) as a white solid (7.65 g, 92%). ^1^H-NMR (400 MHz, DMSO-*d*_6_): δ 10.43 (s, 2H, NH), 3.76 (s, 4H, CH_2_). ^13^C-NMR (100 MHz, DMSO-*d_6_*): δ 161.4, 115.6, 23.9. IR (ATR, cm^−1^): 3201, 3066, 2964, 2923, 1615, 1503, 1392, 1226, 924, 661. Elemental analysis calc. (%) for C_6_H_6_N_4_O_2_ (166.14): C 43.38, H 3.64, N 33.72; found: C 43.35, H 3.75, N 33.61.

*3,7-Diamino-1H,5H-Pyrazolo[1,2-a]Pyrazole-1,5-Dione* (**3**). To a solution of 2-cyano-*N*′-(2-cyanoacetyl)-acetohydrazide (**2**) (4.0 g, 24.07 mmol) in isopropanol (200 mL) neat diethylamine (7.54 mL, 72.22 mmol) was added dropwise at room temperature. At the end of the addition, the reaction mixture was heated at 65 °C for 16 h. After that time, the reaction volume was reduced to about 100 mL by liquid evaporation on a rotary evaporator. The formed precipitate was filtered, washed with isopropanol (5 × 3 mL), and vacuum dried to afford 3,7-diamino-1*H*,5*H*-pyrazolo[1,2-*a*]pyrazole-1,5-dione (**3**) as an off-white solid (3.14 g, 78%). DSC: T_d/onset_ = 257 °C. ^1^H-NMR (400 MHz, DMSO-*d*_6_): δ 7.45 (s, 4H, NH_2_), 4.33 (s, 2H, CH). ^13^C-NMR (100 MHz, DMSO-*d*_6_): δ 165.5, 159.3, 74.8. IR (ATR, cm^−1^): 3392, 3314, 1613, 1572, 1433, 1180, 1092, 772. Elemental analysis calc. (%) for C_6_H_6_N_4_O_2_ (166.14): C 43.38, H 3.64, N 33.72; found: C 43.29, H 3.65, N 33.78.

*3,7-Diamino-2,6-Dinitro-1H,5H-Pyrazolo[1,2-a]Pyrazole-1,5-Dione* (**4**). To a solution of fuming nitric acid (100%, 0.7 mL) in acetic anhydride (2 mL) solid 3,7-diamino-1*H*,5*H*-pyrazolo[1,2-*a*]pyrazole-1,5-dione (**3**) (0.40 g, 2.40 mmol) was slowly added portion wise at 0 °C. At the end of the addition, the reaction mixture was stirred at 0 °C for additional 30 min and then poured into beaker containing ice-water (100 mL). The formed precipitate was filtered, washed with cold water (3 × 10 mL), and vacuum dried to afford 3,7-diamino-2,6-dinitro-1*H*,5*H*-pyrazolo[1,2-*a*]pyrazole-1,5-dione (**4**) as an orange solid (0.56 g, 91%). DSC: T_onset._ = 328.8 °C, T_dec._ = 337 °C (peak). ^1^H-NMR (400 MHz, CCl_3_): δ 9.61 (s, 2H, NH_2_), 9.47 (s, 2H, NH_2_). ^13^C-NMR (100 MHz, CCl_3_): δ 153.5, 151.1, 107.6. IR (ATR, cm^−1^): 3575, 3484, 3349, 3028, 1711, 1660, 1620, 1472, 1386, 1216, 1106. MS (EI): 256 [M]^+^. Elemental analysis calc. (%) for C_6_H_4_N_6_O_6_ (256.13): C 28.14, H 1.57, N 32.81; found: C 28.18, H 1.65, N 32.78.

## 4. Conclusions

A convenient and straightforward four-step synthesis of thermostable 3,7-diamino-2,6-dinitro-1*H*,5*H*-pyrazolo-[1,2-a]pyrazole-1,5-dione (**4**) explosive was developed, starting from cyanoaceto-hydrazide. The structure of compound **4**, containing a new *trans*-bimane explosophore, was comprehensively characterized by various spectroscopic techniques, elemental analysis, and by X-ray crystallography. Compound **4** crystallized as a hydrate, in a tightly packed planar-layered arrangement (with density of 1.845 g·cm^−3^ at 173 K), which is held together by an intramolecular network of hydrogen bonds. Extensive computational studies were conducted to predict the solvent-free crystal structure of compound **4**, which was calculated to have a similar molecular arrangement, but a smaller unit cell volume and, therefore, higher energy density. Compound **4** exhibited impressive thermostability with an onset temperature of decomposition of 328.8 °C, which is on par with known heat-resistant explosives, such as TATB and HNS. These experimental results were in line with our molecular dynamic calculations that predicted close decomposition temperatures of compound **4** and HNS, a reference thermostable explosive. Detonation performance of **4** was calculated by both HASEM and EXPLO 5 (v6.04) software and compared to the reported data for the reference explosives, TATB and HNS, showing that compound **4** has velocity of detonation in the range of 6.88–7.72 km·s^−1^ and detonation pressure in the range of 19.14–24.33 GPa, making **4** a promising explosophore platform for further development of thermostable energetic materials. 

## Figures and Tables

**Table 1:** Calculated and experimental lattice vector lengths, angles, and volume of the primitive cell of compound **4**, along with results from experiments.

	a (Å)	b (Å)	c (Å)	α (°)	β (°)	γ (°)	Volume (Å^3^)
**Calculated**	8.29	9.65	6.41	90.00	93.21	90.00	512.44
**Experimental**	8.22	9.61	6.55	90.00	92.30	90.00	516.65
**Computational error**	0.85%	0.42%	−2.14%	0.00%	0.99%	0.00%	−0.81%
**Solvent-removed**	7.81	9.38	6.32	90.00	94.08	90.00	461.32

**Table 2:** Calculated energetic properties of compound 4 and its isodesmic reaction components

	Compound	Δ_f_H°_gas_ (kJ·mol^−1^)	E_0_ (Hartree)	ZPE (Hartree/Particle)	ΔH_T_ (Hartree/Particle)
1.	**4**		−1010.4391593	0.1334850	0.0151990
2.	CH_4_	−74.8700000	−40.4826803	0.0441530	0.0038100
3.	CH_3_NO_2_	−81.0000000	−244.9469106	0.0491150	0.0052990
4.	CH_3_NH_2_	−23.5000000	−95.8039027	0.0631100	0.0043620
5.	CH_2_O	−115.9000000	−114.4669424	0.0263370	0.0041480
6.	C_6_H_8_N_2_	+423.4445285	−342.6714900	0.1295460	0.0074120

Δ_f_H°_gas_ (C_6_H_8_N_2_) was calculated by G4 method.

**Table 3:** Detonation performance of compound **4**, TATB and HNS calculated by HASEM, for a comparison with the EXPLO 5 (v6.04) calculated results and experimental results.

Compounds and Computation Methods		△H_f_^c^ (kJ·mol^−1^)	VOD (km·s^−1^)	P_d_ (GPa)	Heat of Detonation (kJ·kg^−1^)	Detonation Temp. (K)
**4**	HASEM	−707.18	6.88	22.04	1471.81	2136
	EXPLO 5		7.72	24.33	−3639.187	2810.308
**4-Water Removed**	HASEM		7.02	22.59	2295.46	2787
**TATB**	HASEM	−596.55	7.68	28.17	−3721.24	2155
	EXPLO 5		8.17	28.86	−3876.04	2768
	Experiment		7.76	26.8	−3912.04 [21]	--
**HNS**	HASEM	+173.93	7.34	24.12	−5687.27	4053
	EXPLO 5		7.22	21.98	−4633.30	3444
	Experiment		7.0–7.13 [22]	26.20 [22]	−5690.24 [22]	4150 (other cal.) [23]

**Table 4:** Thermostability and sensitivity properties of compound **4** and other reference explosives.

	Compound	T_d/onset_^a^ (°C)	ρ^b^ (g·cm^−3^)	IS ^c^ (J)	FS ^d^ (N)	ESD ^e^ (J)
1.	**4**	329	1.88	8.23	>360	1.22
2.	**TATB**	330	1.94	50 [24]	>353 [24]	2.5–4.24 [24]
3.	**LLM-105**	298	1.913 [7]	20[25]	360[25]	0.6 [25]
4.	**HNS**	320	1.74	5 [26,27]	240 [26,27]	0.8 [26,27]

^a^ Decomposition temperature (by DSC); ^b^ density (by gas pycnometry, at 25 °C); ^c^ sensitivity to impact (by BAM-fall hammer); ^d^ sensitivity to friction (by BAM-friction tester); ^e^ sensitivity to electrostatic discharge (by OZM-ESD apparatus).

## Supplementary Materials

Figures S1–S4: 1H-NMR spectrum, Figures S5–S8: 13C-NMR spectrum, Figures S9–S12: FTIR spectrum, Table S1: Crystal data and structure refinement for compound **4**, Table S2: Bond Lengths for compound **4**, Table S3: Bond Angles for compound **4**, Table S4: Torsion Angles for compound **4**. Table S5 Hydrogen Atom Coordinates (Å × 104) and Isotropic Displacement Parameters (Å2 × 103) for compound **4**.
