# Supplementary material for: Energetic Butterfly: Heat-Resistant Diaminodinitro trans-Bimane
Source: Molecules. 2019 Nov 26;24(23):4324. doi: 10.3390/molecules24234324 (PMC6930539; doi:10.3390/molecules24234324)
Supplement: Supplementary file 1 [file molecules-24-04324-s001.pdf]

# Supporting Information

# Energetic Butterfly: Heat-Resistant Diaminodinitro *trans*-Bimane

**Pengcheng Zhang [1], Dheeraj Kumar [2,*], Lei Zhang [3], Daniel Shem-Tov [4], Natan Petrutik [4], Ajay Kumar Chinnam [4], Chuang Yao [5], Siping Pang [1,*] and Michael Gozin [4,*]**

1 School of Materials Science and Engineering, Beijing Institute of Technology, Beijing 100081, China; zhangpengchengxyz@163.com
2 Department of Chemistry, Indian Institute of Technology Roorkee, Roorkee, Uttarakhand 247667, India
3 Software Center for High Performance Numerical Simulation, and Laboratory of Computational Physics, Institute of Applied Physics and Computational Mathematics, Beijing 100088, China; zhang_lei@iapcm.ac.cn
4 School of Chemistry, Faculty of Exact Sciences, Tel Aviv University, Tel Aviv 69978, Israel; dstov101@gmail.com (D.S.-T.); petro.natan@gmail.com (N.P.N); ajay0802@gmail.com (A.K.C.)
5 Key Laboratory of Extraordinary Bond Engineering and Advance Materials Technology (EBEAM) of Chongqing, Yangtze Normal University, Chongqing 408100, China; yaochuang@yznu.cn
* Correspondence: dheerajkfcy@iitr.ac.in (D.K.); pangsp@bit.edu.cn (S.P.); cogozin@gmail.com (M.G.); Tel.: +91-1332-285439 (D.K.); +972-364-05878 (M.G.)

**Index**

1. $^{1}$H NMR……………………………………………………………………………………..S2-S5
2. $^{13}$C NMR……………………………………………………………………………………….S6-S9
3. FTIR spectrometry………………………………………………………………………………S10-S11
4. X-ray crystallography……………………………………………………………………………S12-S14

1. **$^{1}$H NMR Spectroscopy**.

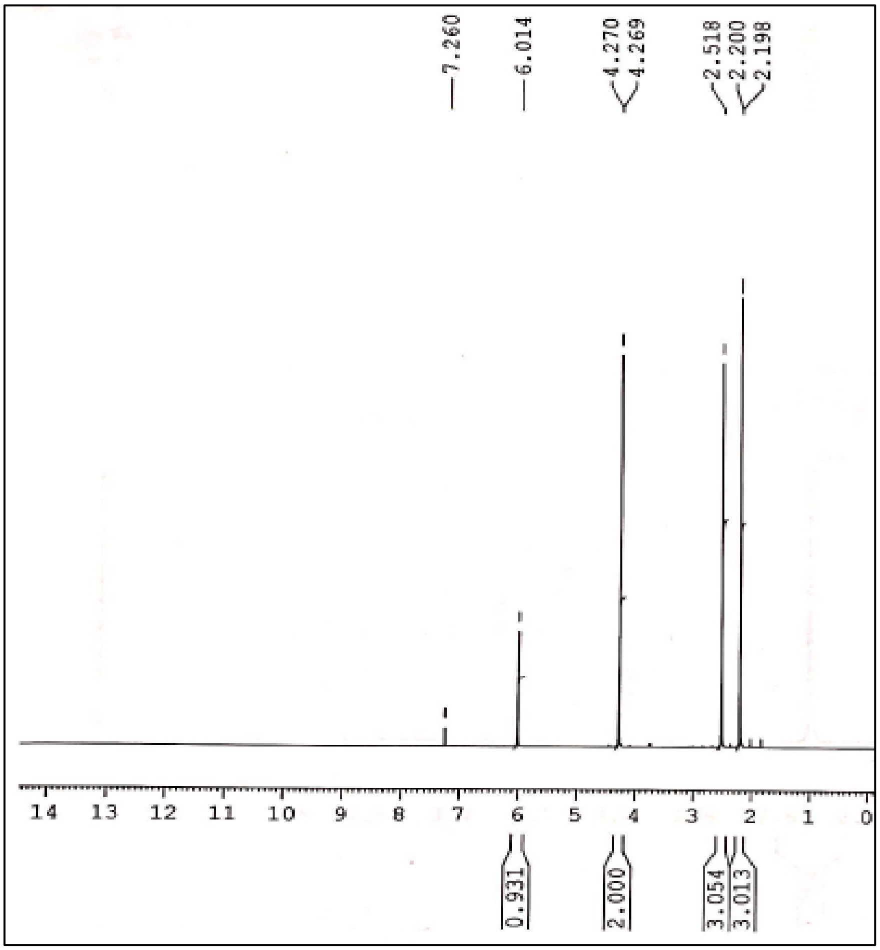


**Figure S1**. $^{1}$H NMR spectrum of compound **1**.

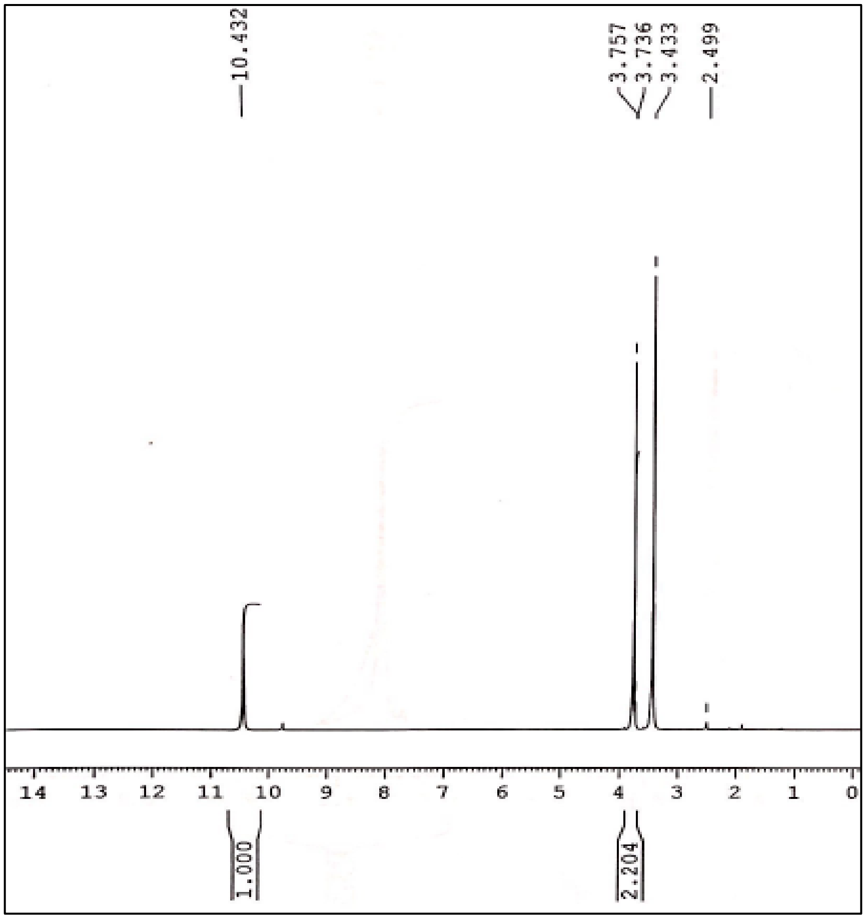


**Figure S2**. $^{1}H$ NMR spectrum of compound **2**.

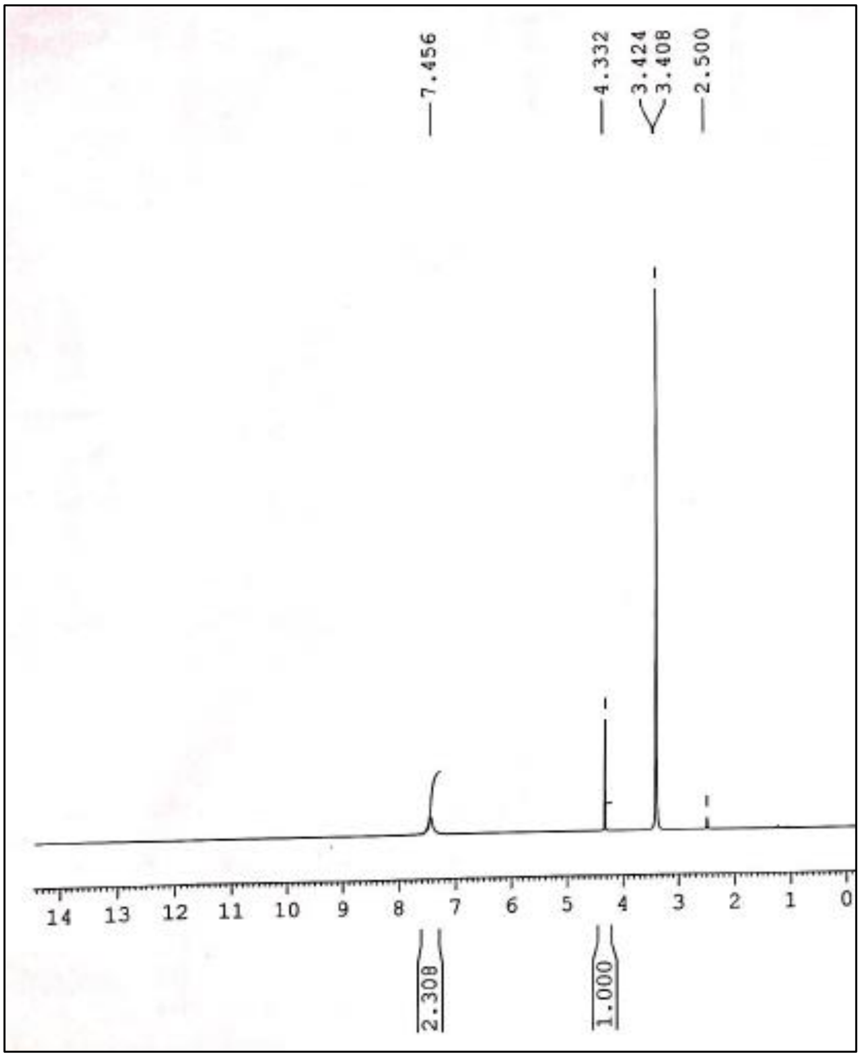


**Figure S3**. $^{1}$H NMR spectrum of compound **3**.

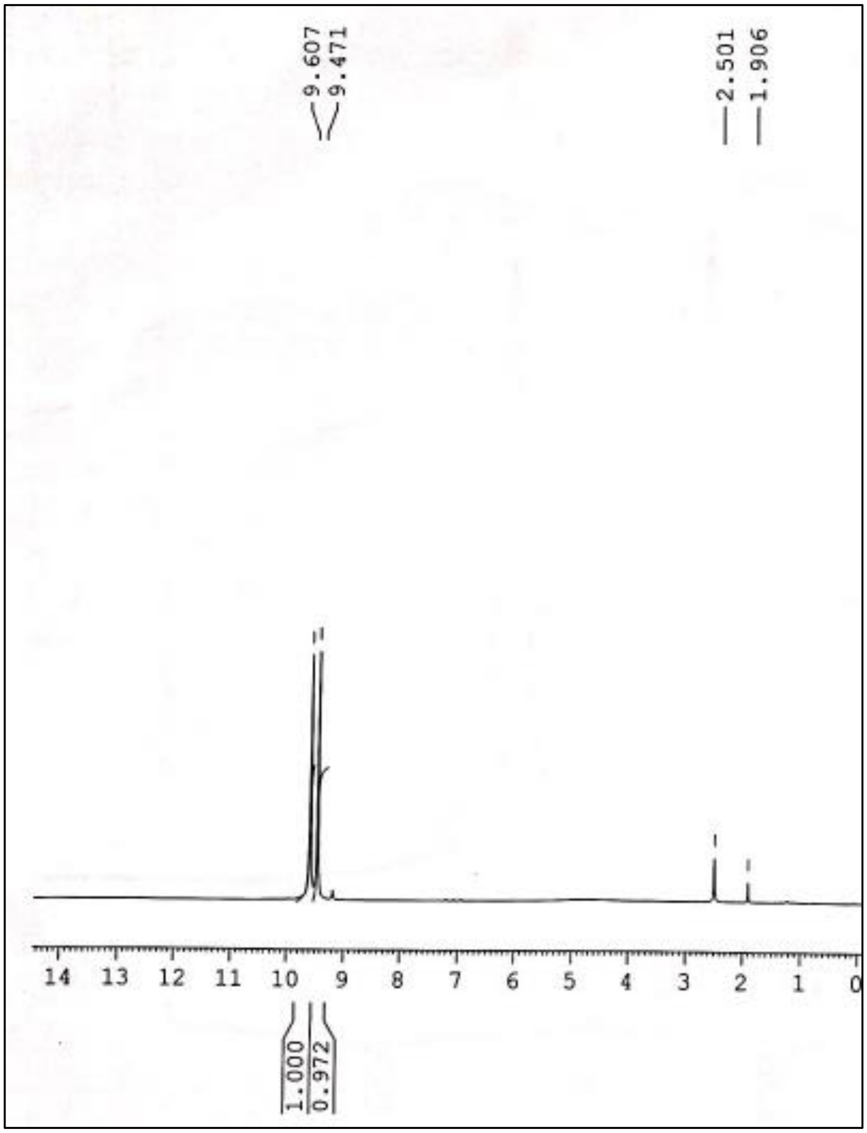


**Figure S4**. $^{1}$H NMR spectrum of compound **4**.

2. **$^{13}$C NMR Spectroscopy**.

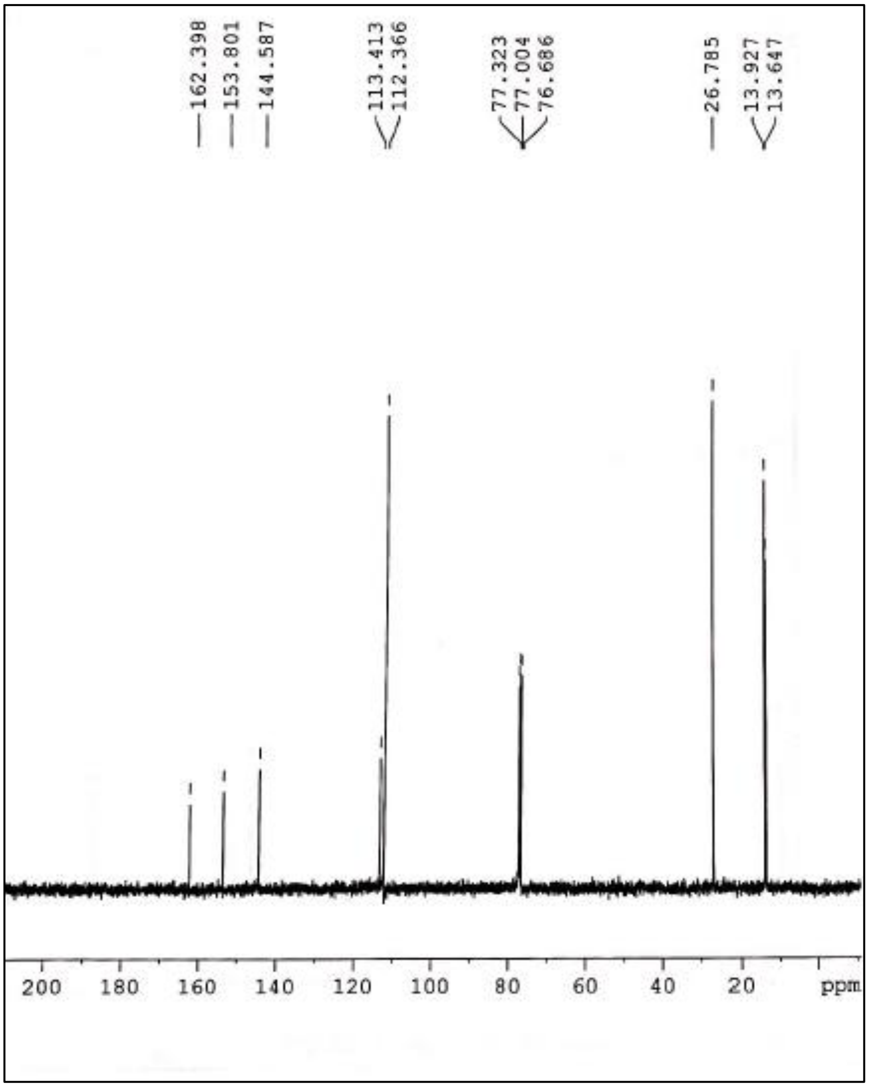


**Figure S5**. $^{13}$C NMR spectrum of compound **1.**

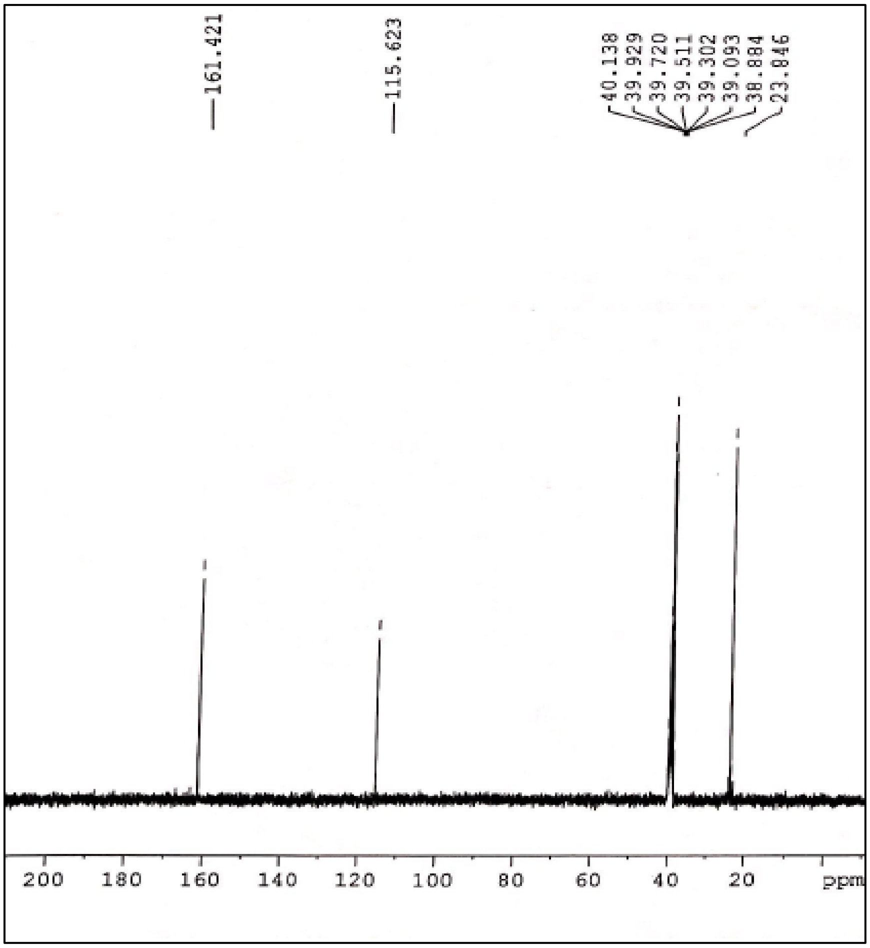


**Figure S6**. $^{13}$C NMR spectrum of compound **2.**

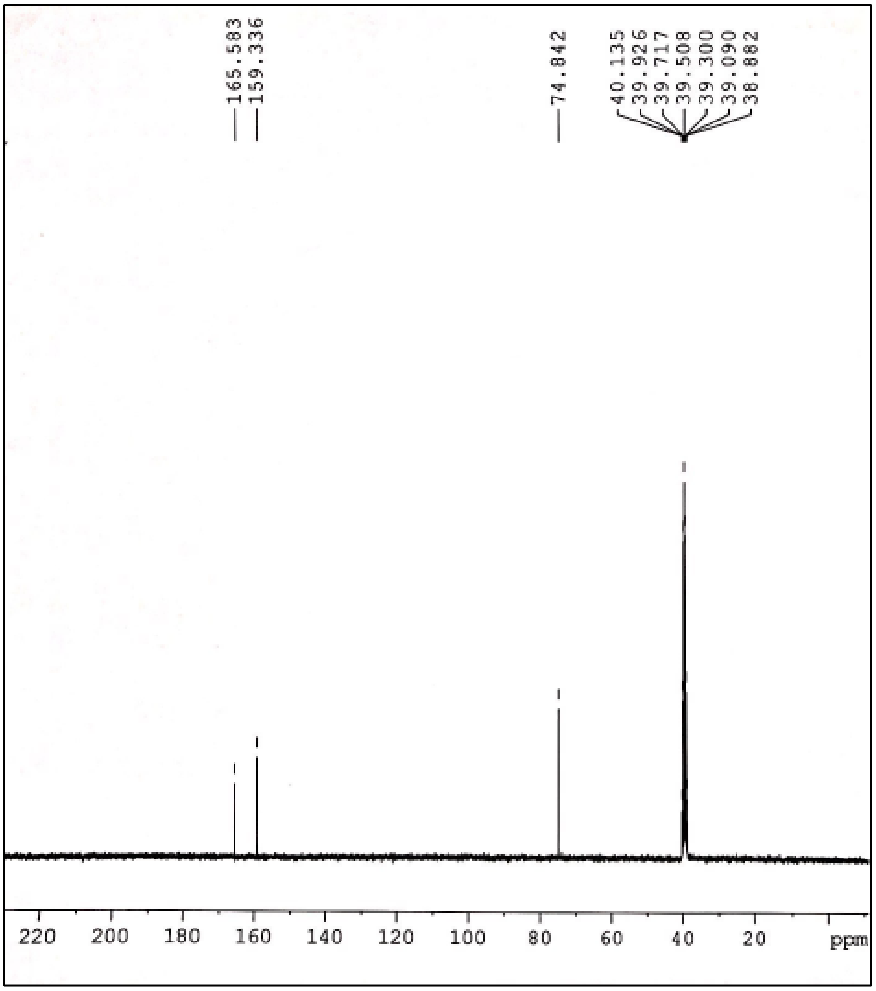


**Figure S7**. $^{13}C$ NMR spectrum of compound **3.**

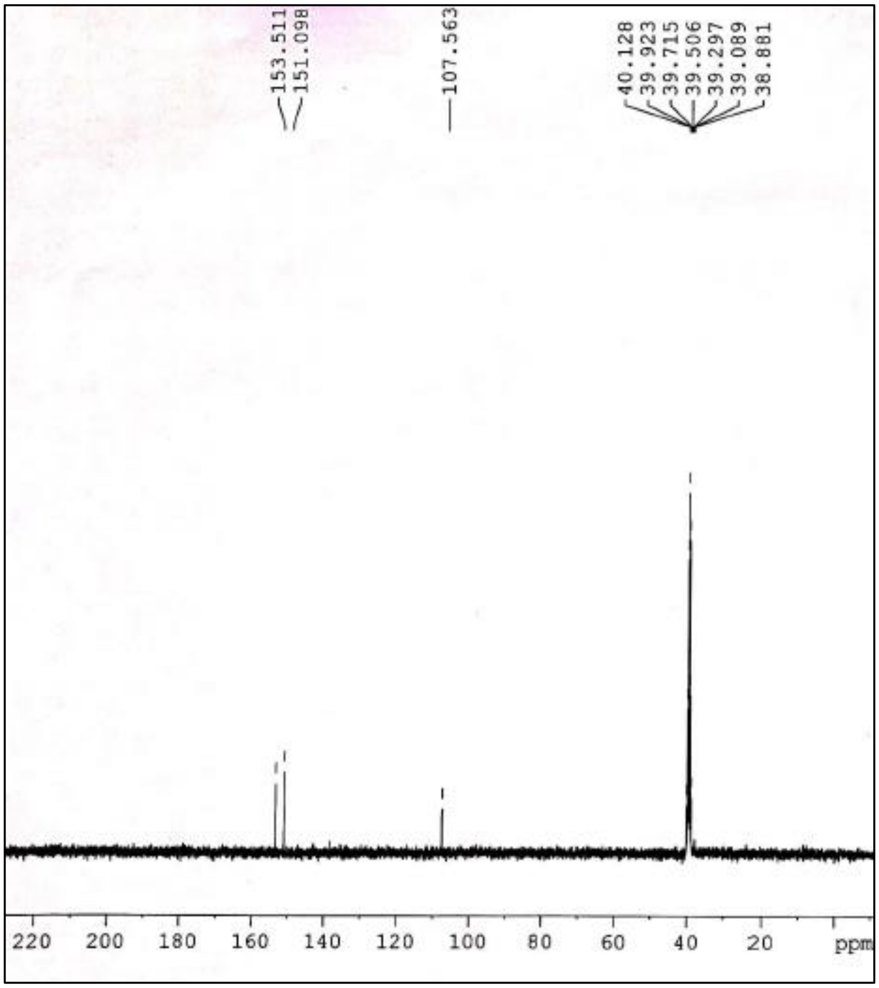


**Figure S8**. $^{13}C$ NMR spectrum of compound **4**.

3. **FTIR Spectroscopy**.

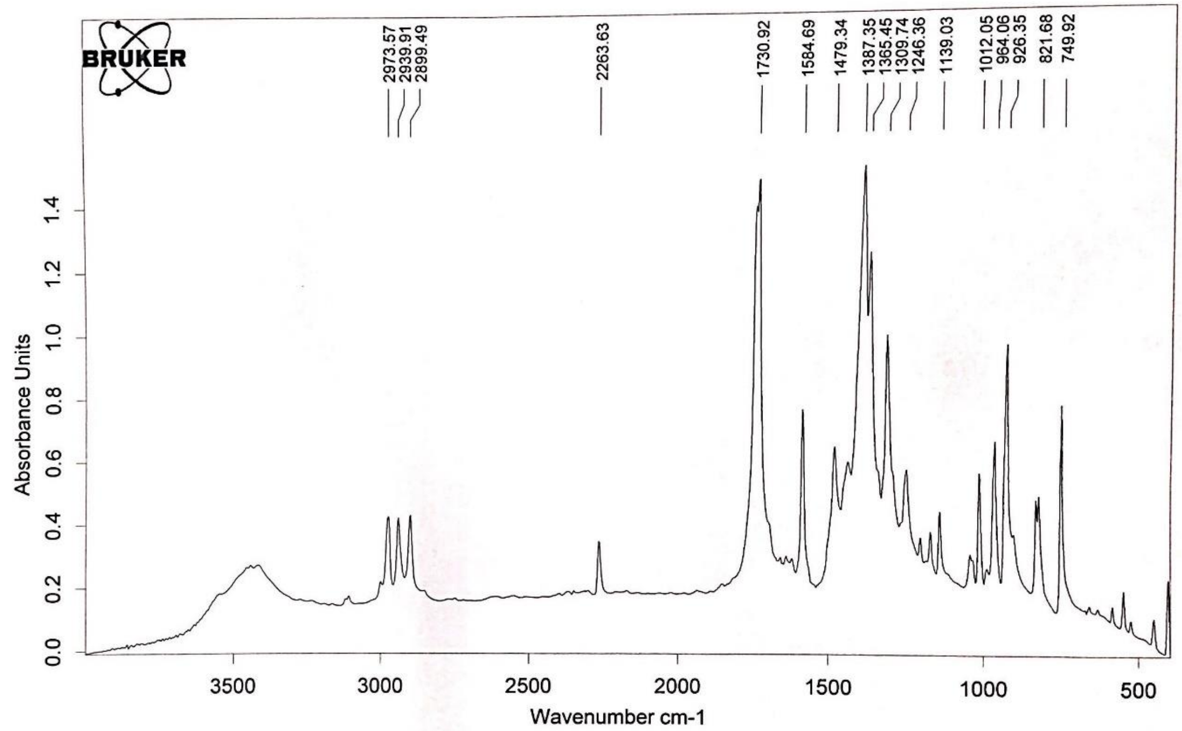


**Figure S9**. FTIR spectra of compound **1.**

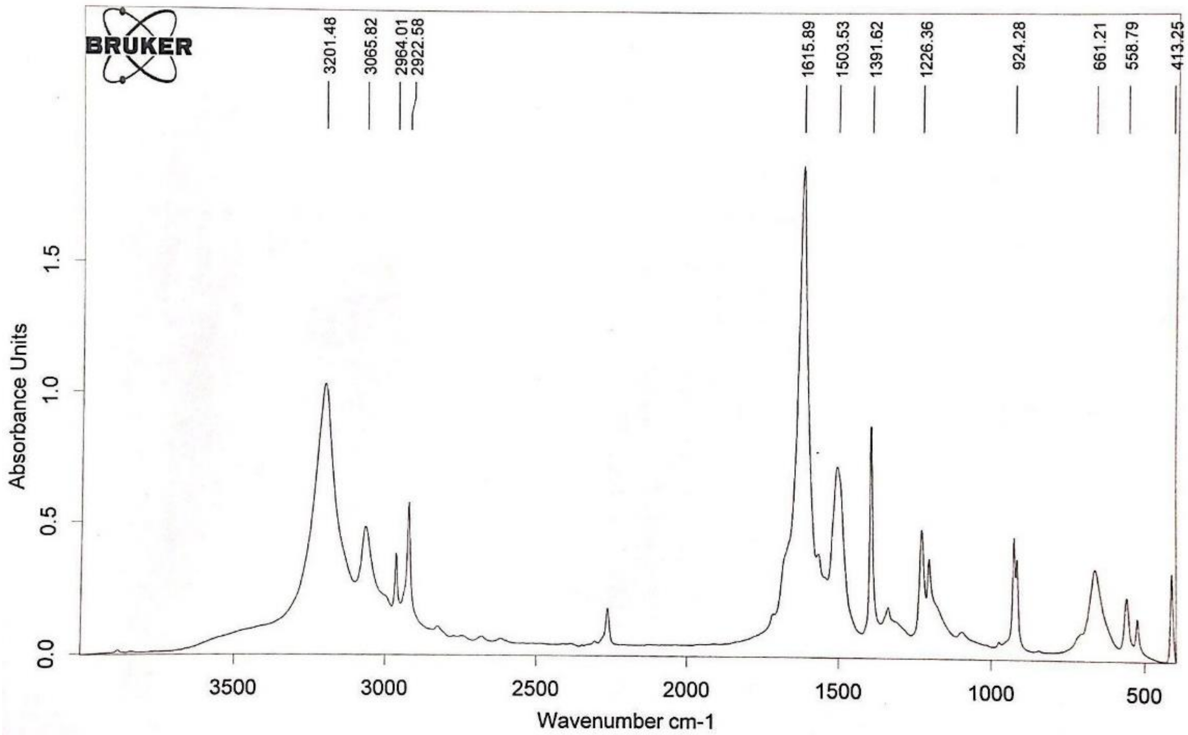


**Figure S10**. FTIR spectra of compound **2.**

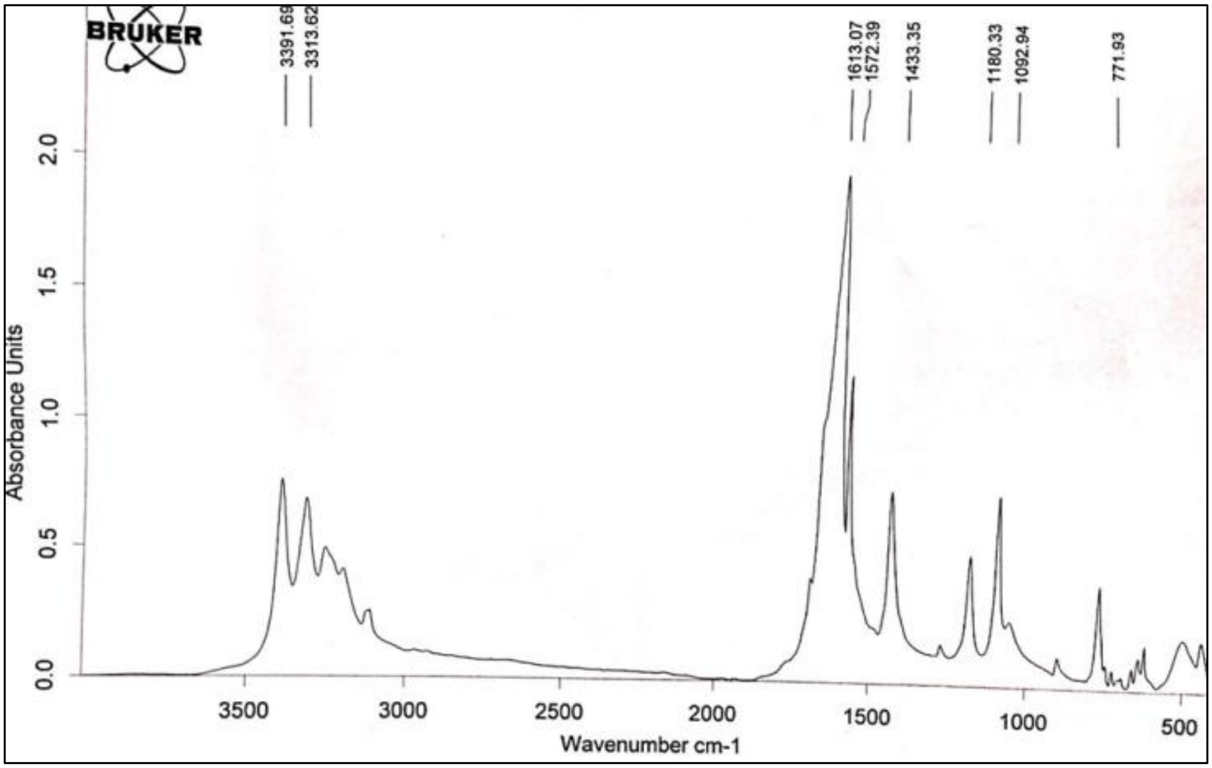


**Figure S11**. FTIR spectra of compound **3.**

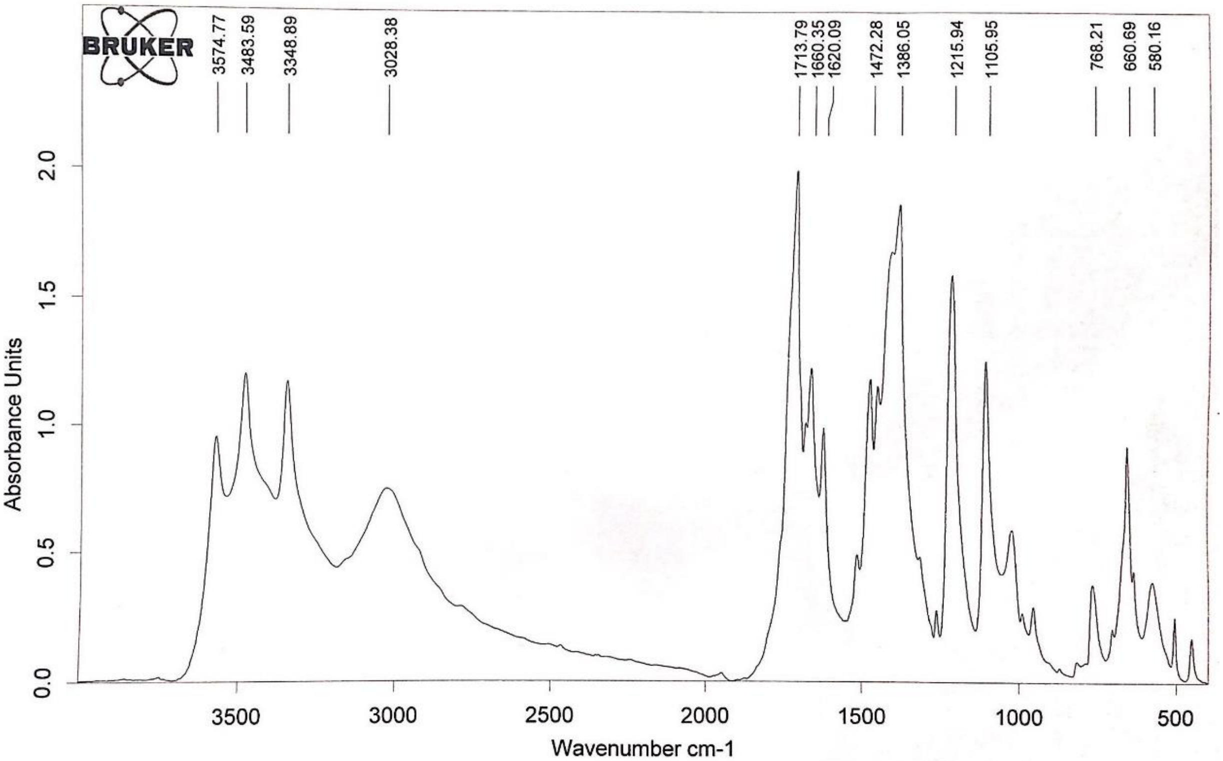


**Figure S12**. FTIR spectra of compound **4.**

## 4. X-ray crystallography data

**Table S1 Crystal data and structure refinement for compound 4.**

| **Parameter** | **4** |
|---|---|
| Formula | $C_6H_4N_6O_6$ |
| *Mr* | 292.18 |
| *T* /K | 173 |
| Crystal system | monoclinic |
| Space group | $P2_1/c$ |
| a/Å | 8.220(2) |
| b/Å | 9.605(2) |
| c/Å | 6.5492(15) |
| a/° | 90 |
| b/° | 92.304(5) |
| g/° | 90 |
| Volume | 516.7(2) |
| Z | 2 |
| $\rho$/g·cm$^{-3}$ | 1.878 |
| Mu/mm$^{-1}$ | 0.175 |
| F(000) | 300 |
| Crystal size/mm3 | 0.18 × 0.11 × 0.08 |
| Radiation | MoKα (λ = 0.71073) |
| $2\theta$ /° range for data collection | 3.525 to 25.561 |
| reflections collected | 1061 |
| Independent reflections | 1061 [$R_{sigma}$ = 0.0200] |
| data/restraints/parameters | 1061/0/94 |
| GOF on $F^2$ | 1.099 |
| $R_1$ [ I>2σ(I)] | 0.0504 |
| $wR_2$ [ I>2σ(I)] | 0.1608 |
| $R_1$ (all data) | 0.0559 |
| $wR_2$ (all data) | 0.1657 |
| largest diff. peak and hole [e Å$^{-3}$] | 0.51/-0.42 |
| CCDC | 1955362 |

**Table S2 Bond Lengths for 4.**

| Atom | Atom | Length/Å |
|---|---|---|
| O1 | N1 | 1.246(2) |
| O2 | N1 | 1.230(2) |
| O3 | C3 | 1.215(3) |
| N1 | C2 | 1.386(2) |
| N2 | C1 | 1.301(3) |
| N4 | N4[1] | 1.390(3) |
| N4 | C1 | 1.359(2) |
| N4 | C3[1] | 1.397(3) |
| C1 | C2 | 1.413(3) |
| C2 | C3 | 1.442(3) |

[1]1-X,1-Y,1-Z

**Table S3 Bond Angles for compound 4.**

| Atom | Atom | Atom | Angle/° |
|---|---|---|---|
| O1 | N1 | C2 | 117.12(18) |
| O2 | N1 | O1 | 122.88(17) |
| O2 | N1 | C2 | 120.00(17) |
| N4[1] | N4 | C3[1] | 110.66(19) |
| C1 | N4 | N4[1] | 109.92(19) |
| C1 | N4 | C3[1] | 139.36(16) |
| N2 | C1 | N4 | 122.02(17) |
| N2 | C1 | C2 | 132.08(17) |
| N4 | C1 | C2 | 105.91(15) |
| N1 | C2 | C1 | 123.86(18) |
| N1 | C2 | C3 | 125.58(19) |
| C1 | C2 | C3 | 110.56(16) |
| O3 | C3 | N4[1] | 122.29(17) |
| O3 | C3 | C2 | 134.80(18) |
| N4[1] | C3 | C2 | 102.91(17) |

[1]1-X,1-Y,1-Z

**Table S4 Torsion Angles for 4.**

| A | B | C | D | Angle/˚ |
|---|---|---|---|---|
| O1 | N1 | C2 | C1 | -0.8(3) |
| O1 | N1 | C2 | C3 | 178.47(18) |
| O2 | N1 | C2 | C1 | 179.19(16) |
| O2 | N1 | C2 | C3 | -1.5(3) |
| N1 | C2 | C3 | O3 | 0.7(3) |
| N1 | C2 | C3 | N41 | -178.93(17) |
| N2 | C1 | C2 | N1 | 0.4(3) |
| N2 | C1 | C2 | C3 | -178.92(18) |
| N41 | N4 | C1 | N2 | 177.98(19) |
| N41 | N4 | C1 | C2 | -1.8(3) |
| N4 | C1 | C2 | N1 | -179.78(16) |
| N4 | C1 | C2 | C3 | 0.8(2) |
| C1 | C2 | C3 | O3 | -179.9(2) |
| C1 | C2 | C3 | N41 | 0.42(18) |
| C31 | N4 | C1 | N2 | 1.2(4) |
| C31 | N4 | C1 | C2 | -178.6(2) |

[1]1-X,1-Y,1-Z

**Table S5 Hydrogen Atom Coordinates (Å×104) and Isotropic Displacement Parameters (Å2×103) for 4.**

| Atom | x | y | z | U(eq) |
|---|---|---|---|---|
| H2A | 2853.5 | 2395.29 | 6029.59 | 29 |
| H2B | 1424.94 | 3258.69 | 6734.24 | 29 |
| H5A | 1840.47 | 96.92 | 6438.64 | 63 |
| H5B | 3278.11 | -346.82 | 5777.08 | 63 |
